# When Ethics Hurts: The Causes and Impact of Moral Distress on Emergency Department Nurses: A Scoping Review

**DOI:** 10.1111/jocn.70391

**Published:** 2026-06-11

**Authors:** C. Walters, M. Murray, C. Young

**Affiliations:** ^1^ Murdoch University Murdoch Western Australia Australia

## Abstract

**Aim:**

To explore the extent and range of published research on the nature of moral distress in emergency nurses.

**Design:**

Scoping review.

**Methods:**

The review followed the Joanna Briggs Institute methodology for scoping reviews and was reported in accordance with the Preferred Reporting Items for Systematic Reviews and Meta‐Analyses Extension for Scoping Reviews. Searches were undertaken with a date limit of 2015–2025. Reference lists of included papers were also screened. Two reviewers independently screened and extracted data, with disagreements resolved through discussion or third‐party review.

**Data Sources:**

Searches were undertaken in August 2025 in CINAHL, MEDLINE, Scopus, and PsycINFO.

**Results:**

Included studies were across diverse international settings with sample sizes ranging from 169 and 390. Thematic analysis identified four overarching themes: factors contributing to moral distress; situational triggers; the impact of moral distress; and interventions and support strategies.

**Conclusion:**

The scoping review identified moral distress as a complex phenomenon that undermines nurse well‐being, reduces professional satisfaction, and compromises care delivery. Common causes include organisational constraints and the depersonalisation of care, with demographic and contextual factors shaping experiences. Most studies focused on describing the experience of moral distress. Few studies considered evaluating interventions or organisational supports. No longitudinal studies were identified, and variation in tools limited comparability. Future research should focus on interventions and longitudinal designs to investigate how moral distress evolves in the emergency nurse population over time and across various stages of practice.

**Implications:**

Establishing evidence‐based strategies to mitigate moral distress is essential for supporting emergency nurses and reducing attrition.

**Impact:**

This study mapped the literature on moral distress in emergency nurses over the last 10 years. It found that most research focuses on measuring moral distress rather than on interventions to prevent or mitigate it. The review will inform nurse leaders, researchers, educators, and policymakers seeking strategies to further support their staff.

**Reporting Method:**

This review adhered to the Joanna Briggs Institute guidelines for scoping reviews.

**Patient or Public Contribution:**

No patient or public contribution.

**Protocol Registration:**

The protocol is registered with Open Science Framework and will be publicly accessible following embargo release in January.

## What Does This Paper Contribute to the Wider Global Community?

- This is the first comprehensive mapping of moral distress research focused on emergency nurses.
- It identifies conceptual and methodological inconsistencies.
- It highlights the need for evidence‐informed and evidence‐based, context‐specific interventions.

## 1 Introduction

Registered Nurses (RNs) are consistently ranked among the most ethical and trusted health professionals, delivering safe, compassionate, and morally sound care (International Council of Nurses 2021). The World Health Organization (2025), however, has estimated a global shortfall of 4.1 million nurses by 2030, noting that significant investment is required to educate and retain the workforce. While there is limited data on how this crisis translates into the emergency nursing context, evidence from Australia suggests the impact is already visible, with more than 300 emergency nursing positions advertised on any given day (Gorman 2019). These workforce challenges are acutely felt in the Emergency Department (ED), a setting characterised by unpredictability, overcrowding, and clinical urgency. In the ED environment, RNs are frequently required to balance competing priorities under significant time pressure.

Working in such conditions has been associated with an increased risk of moral distress, a phenomenon first described by Jameton as the psychological distress experienced when a nurse knows the right course of action but is constrained from acting upon it due to institutional or systemic barriers (Jameton 1984). The understanding of ‘knowing’ reflects a nurse's moral judgement within their professional and ethical framework, acknowledging that what constitutes the right action is shaped by context and individual values. Moral distress has been recognised as a critical issue in nursing ethics, with research identifying its association with burnout, psychological distress, disengagement and attrition from the profession (Austin et al. 2017; Corley et al. 2001; Henrich et al. 2017). Repeated exposure to morally distressing situations may also contribute to moral residue, a lingering emotional burden that affects a nurse's sense of self, moral agency, and professional identity (Epstein and Delgado 2010).

Defining moral distress itself has also been a subject of ongoing debate. Although Jameton's original conceptualisation focused on constraint, subsequent scholars have expanded the definition to include situations where the ethically appropriate course of action is uncertain, contested, or morally ambiguous (Fourie 2015). The construct has further evolved to encompass related concepts such as moral injury and the crescendo effect (Epstein and Hamric 2009), where repeated exposure to moral distress creates an accumulation effect that leads to moral injury, described by Litz et al. (2009, 700) as ‘perpetrating, failing to prevent, bearing witness to, or learning about acts that transgress deeply held moral beliefs and expectations.’ This conceptual diversity has contributed to challenges in measuring and comparing moral distress across studies, with multiple tools and scales employed, often in different contexts or with varying operational definitions. For ED nurses, whose practice sits at the intersection of urgency, uncertainty, and high stakes, clarifying how moral distress is experienced and understood is particularly important for both practice and policy.

Emergency RNs represent a particularly vulnerable cohort for moral distress. The ED environment is distinguished by its need for rapid decision making, patient acuity, unpredictable workloads, and limited continuity of care. Nurses in this setting frequently encounter ethically complex situations, such as delivering perceived futile treatment, managing conflicting wishes between patients and families, and providing care with inadequate resources or staffing (Fernandez‐Parsons et al. 2013; Hou et al. 2021). These conditions amplify the frequency and intensity of moral distress; yet, research specific to ED RNs remains limited. While studies of moral distress in nursing have expanded in recent decades, the evidence is often drawn from Intensive Care Units (ICUs) or general ward environments. Although ICUs are also characterised by high acuity and ethically complex decision making, the nuances of the ED environment differ from the ICU. Nurses working in the ICU typically provide 1:1 or 1:2 care to patients; in contrast, ED nurses manage multiple undifferentiated patients simultaneously, often with limited information and rapidly changing priorities. This lack of ED‐specific research creates a gap in understanding the unique manifestations, causes, and impacts of moral distress in ED RNs.

The consequences of moral distress in ED nursing extend beyond individual wellbeing. At an organisational level, moral distress has been linked to diminished job satisfaction, disengagement, and increased turnover (Austin et al. 2017; Booth et al. 2024). These outcomes contribute to the instability of the workforce, threatening the sustainability of emergency healthcare delivery at a time when demand for emergency care rises globally post‐COVID‐19. Unresolved moral distress has been shown to negatively impact the quality and safety of patient care, as nurses experiencing moral distress have been reported to have higher levels of compassion fatigue (Ahmadi et al. 2025; Albaqawi and Alshammari 2025). Addressing moral distress in ED RNs is not only an important consideration from a wellness perspective but also a matter of workforce sustainability and patient safety.

The Moral Distress Scale (MDS) was originally developed by Corley et al. (2001) to measure moral distress in ICU nurses and focuses on institutional constraints as the root causes of moral distress. The Moral Distress Scale‐Revised (MDS‐R) developed by Hamric et al. (2012) moved away from the ICU focus to enhance applicability to other health professions and areas of practice and has a more explicit definition of moral distress. The MDS‐R included three root causes of moral distress, these being clinical situations, internal constraints, and external constraints (Hamric et al. 2012). While the MDS‐R built on the earlier work of Corley et al. (2001), there are multiple different versions of the scale for different areas of practice; for example, paediatrics. Epstein et al. (2019) expanded this work and developed a single scale applicable across all health professions and clinical settings: the Measure of Moral Distress for Health Professionals (MMD‐HP). In the development, Epstein et al. (2019) identified key components of moral distress: complicity in wrongdoing, lack of voice, wrongdoing associated with professional (not personal) values, and repeated exposure to morally distressing situations. Furthermore, the three levels of root causes previously identified by Hamric et al. (2012) were clarified to become the patient level, unit level, and system level. As a result, these instruments do not measure an equivalent construct, complicating interpretation of moral distress across studies and contexts.

Despite these concerns, the literature concerning moral distress of RNs working in the ED is fragmented. Previous research has explored moral distress in nursing more broadly, including critical care RNs; however, few have focused specifically on the ED RN context. Given the varied conceptual definitions and tools for measuring moral distress, a scoping review is well‐suited to capture the breadth and heterogeneity of this evidence. The overarching objective of this scoping review is to map and synthesise the existing evidence on moral distress among emergency RNs to discover what is known about the experience and impact of moral distress in ED RNs and identify if there are any common causes of moral distress in this population.

## 2 The Review

### 2.1 Aim

This scoping review aims to explore the extent, range, and nature of published research on moral distress in emergency nurses.

### 2.2 Methods

#### 2.2.1 Design

This scoping review was conducted in accordance with the methodological framework originally proposed by Arksey and O'Malley (2005) and subsequently refined by Levac et al. (2010). This framework follows the best practice guidelines set out in the JBI Manual for Evidence Synthesis: Scoping Review Chapter (Peters et al. 2020). This approach ensures a comprehensive process with methodological rigour. The scoping review comprised five stages, as described by Levac et al. (2010).

##### 2.2.1.1 Stage 1: Identifying the Research Question

The research questions were developed using the Population, Concept, Context (PCC) framework. The population was Registered Nurses, the concept of interest was moral distress, and the context was the ED. The aim of the review was to explore the experience of moral distress among ED nurses, its impact on this population, and to identify common causes. The specific research questions were:

- What is known about the experience and impact of moral distress among emergency nurses?
- What are the reported causes of moral distress in emergency nurses?

##### 2.2.1.2 Stage 2: Identification of Studies

###### 2.2.1.2.1 Inclusion Criteria

Inclusion criteria were established according to the PCC framework. Eligible studies included RNs working in EDs. For the purpose of this study, Emergency Departments were defined as ‘A 24‐h department within a hospital for diagnosis and treatment of acute and urgent illnesses, where patients are seen in order of urgency’ (Independent Health and Aged Care Pricing Authority 2024). Studies of other disciplines were only included in the data if ED nurse data were presented separately. Studies were eligible if they investigated the experience, causes, or consequences of moral distress among ED nurses. Studies set in critical care, community‐based, or primary care settings were excluded unless data specific to the ED could be extracted. Only peer‐reviewed primary research using quantitative, qualitative, or mixed methods designs was included. Systematic reviews, literature reviews, commentaries, editorials, and conference abstracts were excluded. Publications were limited to English language articles from 2015 to 2025.

A comprehensive search strategy was developed and tested in May 2025 and updated in July and August 2025. The following electronic databases were searched: CINAHL Ultimate, MEDLINE (OVID), APA PsycINFO, and Scopus. The search combined headings (e.g., MeSH) and keywords related to moral distress and emergency nursing, adapted for each database. Full search strategies are presented in Table 1. Reference lists of included studies were hand‐searched to identify additional material. All results were imported into EndNote (version 20) for deduplication and then transferred to Covidence (Veritas Health Innovation 2025) for screening and data management.

**TABLE 1:** Database search terms.

Database	Search (S) terms
CINAHL	S1 – "Moral Distress" OR "Ethical Dilemmas" OR "Ethics, Nursing" OR moral distress OR ethical conflict OR ethical dilemma* OR moral conflict OR moral injury S2 – "Emergency Nurses" OR "Emergency Nursing" OR "Emergency Service, Hospital" OR emergency nurse* OR ED nurse* OR A&E nurse* OR ER nurse* OR emergency department nurs* OR emergency room nurs* S3 – experience* OR perception* OR attitude* OR impact OR psychological impact OR emotional burden OR job satisfaction OR burnout OR retention S4 – S1 AND S2 S5 – S1 AND S3 S6 – S1 AND S2 AND S3 S7 – S2 AND S3
MEDLINE (OVID)	S1 – "Moral Distress" OR "Ethical Dilemmas" OR "Ethics, Nursing" OR moral distress OR ethical conflict OR ethical dilemma* OR moral conflict OR moral injury S2 – S1 limited to Abstracts, English, Human, Full text, 2015‐current S3 – "Emergency Nurses" OR "Emergency Nursing" OR "Emergency Service, Hospital" OR emergency nurse* OR ED nurse* OR A&E nurse* OR ER nurse* OR emergency department nurs* OR emergency room nurs* S4 – S3 limited to Abstracts, English, Human, Full text, 2015‐current S5 – experience* OR perception* OR attitude* OR impact OR psychological impact OR emotional burden OR job satisfaction OR burnout OR retention S6 – S5 limited to Abstracts, English, Human, Full text, 2015‐current S7 – S2 AND S4 S8 – S2 AND S6 S9 – S2 AND S4 AND S6 S10 – S4 AND S6
Apa PsychINFO	S1 – "Moral Distress" OR "Ethical Dilemmas" OR "Ethics, Nursing" OR moral distress OR ethical conflict OR ethical dilemma* OR moral conflict OR moral injury S2 – "Emergency Nurses" OR "Emergency Nursing" OR "Emergency Service, Hospital" OR emergency nurse* OR ED nurse* OR A&E nurse* OR ER nurse* OR emergency department nurs* OR emergency room nurs* S3 – experience* OR perception* OR attitude* OR impact OR psychological impact OR emotional burden OR job satisfaction OR burnout OR retention S4 – S1 AND S2 S5 – S1 AND S3 S6 – S1 AND S2 AND S3 S7 – S2 AND S3
Scopus	S1 – "Moral Distress" OR "Ethical Dilemmas" OR "Ethics, Nursing" OR moral distress OR ethical conflict OR ethical dilemma* OR moral conflict OR moral injury AND (LIMIT‐TO (SUBJAREA, "MEDI") OR LIMIT‐TO (SUBJAREA, "NURS") OR LIMIT‐TO (SUBJAREA, "PSYC") OR LIMIT‐TO (SUBJAREA, "HEAL")) AND (LIMIT‐TO (DOCTYPE, "ar")) AND (LIMIT‐TO (EXACTKEYWORD, "Human") OR LIMIT‐TO (EXACTKEYWORD, "Humans")) AND (LIMIT‐TO (SRCTYPE, "j")) AND (LIMIT‐TO (LANGUAGE, "English")) S2 – "Emergency Nurses" OR "Emergency Nursing" OR "Emergency Service, Hospital" OR emergency nurse* OR ED nurse* OR A&E nurse* OR ER nurse* OR emergency department nurs* OR emergency room nurs* AND (LIMIT‐TO (SUBJAREA, "MEDI") OR LIMIT‐TO (SUBJAREA, "NURS") OR LIMIT‐TO (SUBJAREA, "HEAL")) AND (LIMIT‐TO (DOCTYPE, "ar")) AND (LIMIT‐TO (EXACTKEYWORD, "Human") OR LIMIT‐TO (EXACTKEYWORD, "Humans")) AND (LIMIT‐TO (SRCTYPE, "j")) AND (LIMIT‐TO (LANGUAGE, "English")) S3 – experience* OR perception* OR attitude* OR impact OR psychological impact OR emotional burden OR job satisfaction OR burnout OR retention AND (LIMIT‐TO (SUBJAREA, "MEDI") OR LIMIT‐TO (SUBJAREA, "PSYC") OR LIMIT‐TO (SUBJAREA, "NURS") OR LIMIT‐TO (SUBJAREA, "HEAL")) AND (LIMIT‐TO (EXACTKEYWORD, "Human") OR LIMIT‐TO (EXACTKEYWORD, "Humans")) AND (LIMIT‐TO (SRCTYPE, "j")) AND (LIMIT‐TO (LANGUAGE, "English")) S4 – S1 AND S2 S5 – S1 AND S3 S6 – S1 AND S2 AND S3 S7 – S2 AND S3

##### 2.2.1.3 Stage 3: Study Selection

Titles and abstracts were screened in Covidence by two reviewers (CW and MM) against eligibility criteria. Full texts were then independently assessed for inclusion, with disagreements resolved through discussion and, if necessary, consultation with a third reviewer (CY). The process was iterative, with refinements made as familiarity with the evidence base increased. For example, studies initially identified under broader terms, such as ‘critical care’, were excluded if not directly related to ED nursing. The Preferred Reporting Items for Systematic reviews and Meta‐Analyses extension for Scoping Reviews (PRISMA‐ScR) checklist guided reporting. The PRISMA‐ScR flow diagram (Figure 1) illustrates the screening and selection process.

**FIGURE 1:**
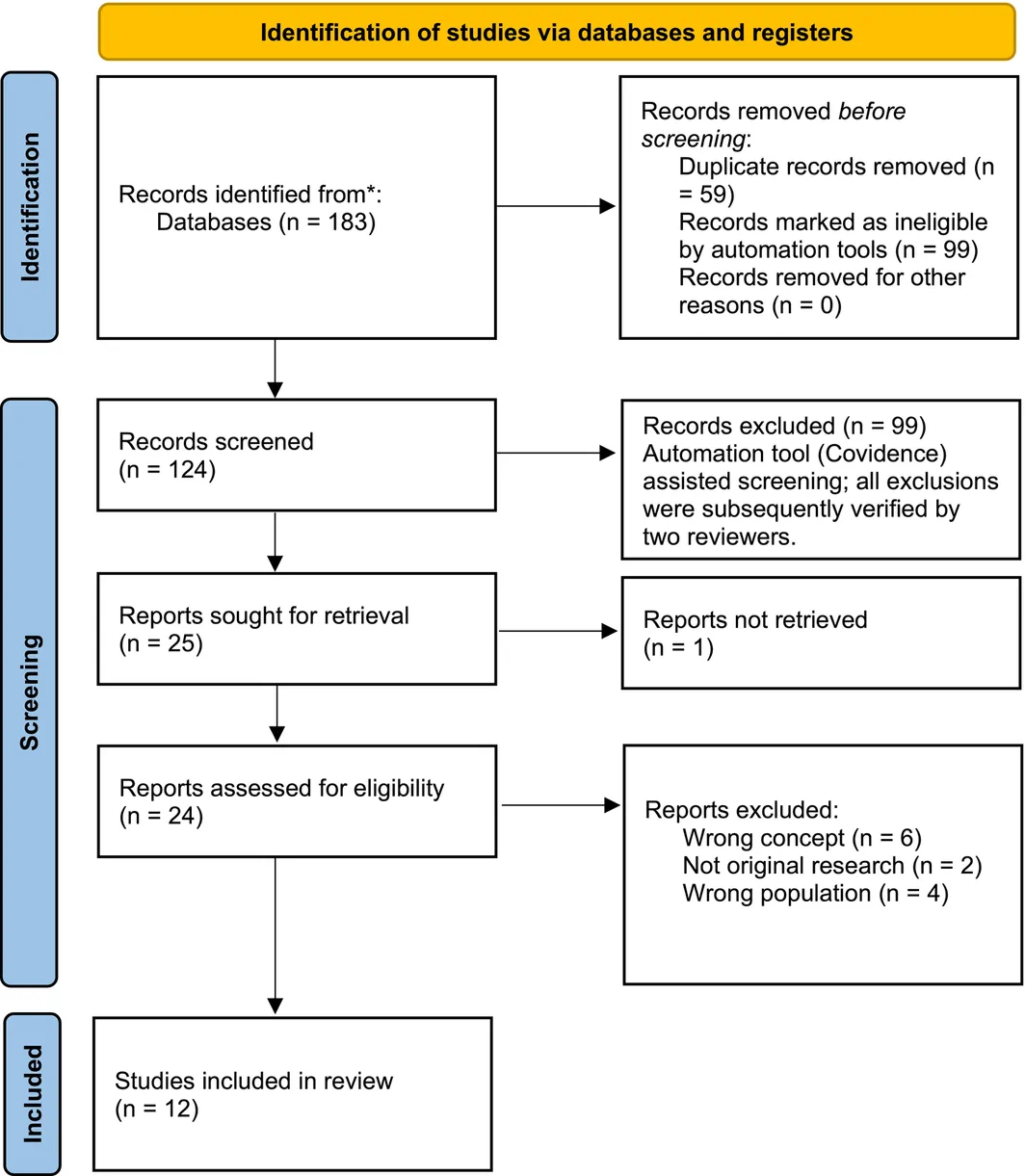
Preferred Reporting Items for Systematic reviews and Meta‐Analyses extension for Scoping Reviews (PRISMA‐ScR). [Colour figure can be viewed at wileyonlinelibrary.com]

##### 2.2.1.4 Stage 4 Charting the Data

Data from included studies were charted in NVivo using a structured extraction framework. Study characteristics (authorship, year, country, design, sample size, population, setting, and measurement tools) were recorded in case classifications to enable mapping across the evidence base. Conceptual details were then coded to categories aligned with the review questions, including experience, causes, impacts, and interventions related to moral distress. Additional codes were created when new themes emerged. Charting was treated as an iterative process, with categories refined as familiarity with the literature increased, allowing both numerical mapping of study characteristics and thematic organisation of study findings.

##### 2.2.1.5 Stage 5 Collating, Summarising and Reporting the Results

Data were collated and analysed in NVivo using both descriptive and thematic approaches. A summary of study characteristics (country, year, design, population and key findings) was produced to map the extent and distribution of research activity (Table 2). Thematic coding enabled comparison of findings across studies in relation to the review questions. Results are presented in tabular format, outlining key strengths and gaps in the evidence base. This approach supports both mapping of the field and identification of priorities for future research and practice.

**TABLE 2:** Summary table of study characteristics.

Citation details (Author, Year, Country)	Study design	Sample	Population	Measurement tools	Key findings
Babamohamadi et al. (2021) Moral distress and its contributing factors among emergency department nurses: A cross‐sectional study in Iran Iran	Descriptive cross‐sectional	203 Single site	RN	MDS‐R	ED nurses in Iran reported moderate levels of moral distress, with the most severe causes related to initiating life‐saving interventions perceived as futile. Moral distress was significantly associated with younger age and fewer years of clinical experience. Data collected pre‐COVID‐19
Clark et al. (2021) Resilience, Moral Distress, and Workplace Engagement in Emergency Department Nurses. USA	Cross‐section, correlational.	173 Five hospital in two states Six social media groups of a professional nursing organisation in two states	RN, Licensed Practical Nurses (LPN), Advanced Practice Registered Nurses (APRN)	Connor‐Davidson Resilience Scale Measure of Moral Distress for Healthcare Professionals Utrecht Workplace Engagement Scale	Emergency nurses experienced moderate moral distress, which was negatively associated with workplace engagement and positively associated with job dissatisfaction and intent to leave. Resilience was related to engagement but did not directly correlate with moral distress Data collected pre‐COVID‐19
Clark et al. (2022) Resilience, Moral Distress, and Job Satisfaction Driving Engagement in Emergency Department Nurses A Qualitative Analysis. USA	Qualitative Semi‐structured interviews	15 Five hospital in two states Six social media groups of a professional nursing organisation in two states	ED nurses	Theoretical qualitative content analysis	Moral distress arose primarily from system‐level constraints (staffing shortages, poor teamwork, limited autonomy) and contributed to reduced engagement and withdrawal behaviours. Supportive leadership, recognition, and teamwork mitigated moral distress and enhanced engagement. Data collected pre‐COVID‐19
Jalali et al. (2019) Moral Distress and Related Factors Among Emergency Department Nurses Iran	Analytic‐descriptive cross‐sectional	180 Single site	RN	‘Jamitoon’ moral distress scale	Emergency nurses reported high levels of moral distress, particularly when providing care in unsafe or understaffed conditions. Barriers to professional development and inadequate resources were key predictors, highlighting organisational contributors to distress. Data collected pre‐COVID‐19
Jiménez‐Herrera and Axelsson (2015) Some ethical conflicts in emergency care. Spain	Qualitative Semi‐structured interview guide Focus group discussion guide (based on interview themes)	16 Single site	RN (ED and pre hospital nurses)	Qualitative content analysis framework (Mayring's method)	Moral distress emerged from ethical conflicts related to autonomy, depersonalisation of patients, inadequate pain management, and end‐of‐life care. Power imbalances and biomedical dominance limited holistic nursing practice, contributing to sustained ethical tension. Data collected pre‐COVID‐19
Kızıltepe and Koç (2025) Exploring Moral Distress, Related Factors and Coping in Emergency Nurses: A Mixed Method Study Turkey	Mixed methods	252 Quantitative 23 Qualitative 18 hospitals in one region	RN	Moral distress scale (MDS) COPE Scale Demographic information	Emergency nurses experienced disturbing levels of moral distress, driven by incompetent colleagues, unsafe staffing, cost pressures, and poor communication. Moral distress was associated with burnout, guilt, and reduced motivation, with limited organisational support despite individual coping efforts. Data collected pre‐COVID‐19
Li et al. (2024) Thriving at work, career calling, and moral distress among nurses. China	Quantitative – cross‐sectional	390 National	RN	Thriving at Work Scale Career Calling Scale Moral Distress Scale‐ Revised.	Moral distress was indirectly influenced by thriving at work, operating through career calling as a full mediator. Stronger career calling was associated with lower moral distress, suggesting professional meaning buffers distress rather than work thriving alone. Data collection year not specified. No reference to COVID‐19 within results or discussion.
Robinson and Stinson (2016) Moral Distress A Qualitative Study of Emergency Nurses USA	Qualitative Phenomenological Structured open ended interviews	8 Three sites	RN	Thematic analysis	Moral distress was described as frequent and intense, shaped by futile care, lack of family advocacy, and value conflict. Nurses reported emotional suppression, moral residue, identity changes, burnout, and workforce exit, underscoring cumulative harm. Data collected pre‐COVID‐19
Topan et al. (2023) Moral Distress and Its Relating Factors Among Paediatric Emergency Department Nurses A Cross‐Sectional Study Turkey	Quantitative Descriptive Cross‐sectional	255 Multi‐site in one region	RN	MDS‐R (paediatric version)	Paediatric emergency nurses reported generally low but variable moral distress, higher than general ED contexts when caring for children. Greater distress was associated with low job satisfaction, higher education, longer hours, and unnecessary treatments ordered by physicians. Data collected in 2022. No reference to COVID‐19 within results or discussion.
Trautmann et al. (2015) Relationships Among Moral Distress, Level of Practice Independence, and Intent to Leave of Nurse Practitioners in Emergency Departments Results From a National Survey. USA	Quantitative Cross‐sectional	207 National	Nurse Practitioners (NP)	MDS‐R Dempster Practice Behaviour Scale	Emergency nurse practitioners experienced moderate moral distress, primarily related to poor communication, unsafe staffing, and incompetent colleagues. Moral distress significantly predicted intent to leave, while practice independence showed no protective effect. Data collected pre‐COVID‐19
Zavotsky and Chan (2016) Exploring the Relationship Among Moral Distress, Coping, and the Practice Environment in Emergency Department Nurses. USA	Descriptive correlational	198 Single site	RN	MDS‐R COPE	Emergency nurses reported moderate moral distress, commonly triggered by futile care, unsafe staffing, and following unnecessary physician orders. Supportive practice environments were associated with lower distress and reduced turnover intentions, highlighting organisational influence. Data collected pre‐COVID‐19
Ziba et al. (2022) The Relationship between Health Anxiety and Perceived Stress with Moral Distress in Emergency Nurses: The Mediating Role of Distress Tolerance Iran	Descriptive‐correlational	230 Multi‐site, one city	Emergency nurses	Health Anxiety Questionnaire Perceived Stress Questionnaire Distress Tolerance Scale Jameton Moral Perturbation Questionnaire	Moral distress was positively associated with perceived stress and health anxiety, and negatively associated with distress tolerance. Distress tolerance mediated these relationships, suggesting psychological resilience as a potential intervention target. Data collected during the pandemic (2020), however, there is no discussion surrounding the impact of the pandemic on moral distress on the results for the moral distress aspect of this article.

### 2.3 Data Analysis

Extracted data were collated and summarised in tabular form for numerical summary and qualitative thematic analysis. Data synthesis examined similarities and differences in terms of approach, including methodology, theoretical frameworks, tools, and definitions of moral distress. Focus was paid to the detail surrounding the experience, impact, and causes of moral distress in emergency nurses.

## 3 Results

The database searches returned a large number of records for the initial concept sets. When concepts were combined using Boolean operators AND or OR, the number of potentially relevant articles was reduced. Across all databases, 183 records were exported to Covidence (Veritas Health Innovation 2025) for screening. After the removal of 59 duplicates, 124 unique records remained and were assessed at title and abstract level. Of these, 99 were excluded as irrelevant, leaving 25 articles for full text review. Following the full text assessment, 13 further articles were excluded for the following reasons: wrong concept (*n* = 6), not original research (*n* = 2), wrong population (unable to extrapolate ED or ED RN data; *n* = 4), and unable to access full text (*n* = 1). Twelve studies met the inclusion criteria and were included in the final review.

### 3.1 Key Themes/Findings

#### 3.1.1 Characteristics of Included Studies

All included studies were published between 2015 and 2025 and conducted across diverse international contexts including the United States of America (USA; *n* = 5), Iran (*n* = 3), Turkey (*n* = 2), and single studies from Spain and China. Most studies used quantitative cross‐sectional designs (*n* = 7) with sample sizes ranging from 169 to 390 nurses. Qualitative studies (*n* = 3) and mixed‐methods studies (*n* = 2) provided further depth to the accounts of emergency RN experiences. The Moral Distress Scale—Revised (MDS‐R) was the most frequently used tool (*n* = 5), with the original Moral Distress Scale (MDS) used second most frequently (*n* = 2), and the Measure of Moral Distress—Health Professionals (MMD‐HP) used the least frequently, along with the Moral Perturbation Questionnaire (*n* = 1). The remaining three studies were qualitative and used a mixture of semi‐structured and structured open‐ended interviews and focus groups.

The included studies varied widely in design, sample size, data collection approaches and tools, complicating methodological heterogeneity. This methodological heterogeneity limited the comparability of findings across studies and limits the extent to which patterns of moral distress can be consistently interpreted. The majority of quantitative studies (Clark et al. 2021; Li et al. 2025; Trautmann et al. 2015; Zavotsky and Chan 2016; Ziba et al. 2022) recruited participants through convenience sampling, identified by Sandelowski (1995) as the least rigorous of sampling methods, but required when identifying specific samples of participants (Braun and Clarke 2013). Two quantitative studies (Babamohamadi et al. 2021; Jalali et al. 2019) are both from Iran and used a more rigorous census sampling process, which may limit the generalisability of findings to Western ED settings, while one study (Topan et al. 2023) from Turkey used a snowball sampling technique. Furthermore, there are contextual variations between countries represented, with health‐care systems, scope of practice, staffing models, and cultural influences potentially impacting causes of moral distress. Factors identified as a source of moral distress in one system may not be transferrable to others.

The predominance of cross‐sectional designs (*n* = 8) limits understanding of how moral distress develops, fluctuates, or resolves over time. These studies capture moral distress at a single time point (Liamputtong 2017), restricting temporal ordering and precluding causal inference. Consequently, it remains unclear whether moral distress precedes outcomes such as burnout or disengagement, or whether these outcomes increase vulnerability to moral distress. No longitudinal studies were identified.

While eight studies employed multi‐site sampling (Clark et al. 2021, 2022; Kızıltepe and Koç 2025; Li et al. 2024; Robinson and Stinson 2016; Topan et al. 2023; Trautmann et al. 2015; Ziba et al. 2022), the scope and configuration of sites varied substantially, ranging from national surveys (Li et al. 2025; Trautmann et al. 2015) to single sites (Babamohamadi et al. 2021; Jalali et al. 2019; Jiménez‐Herrera and Axelsson 2015; Zavotsky and Chan 2016). In two studies (Topan et al. 2023; Ziba et al. 2022), the number and characteristics of the participating sites were poorly specified. This heterogeneity limits cross‐study comparison and complicates interpretation of moral distress prevalence and causes, as organisational context and ethical climate may differ across emergency care settings.

#### 3.1.2 Findings

Thematic analysis (Braun and Clarke 2013) was used to identify patterns across the included studies. Four overarching themes emerged in relation to the causes, experience and impact of moral distress on ED RNs, these were: factors shaping the experience of moral distress; situational triggers of moral distress in emergency nurses; the impact of moral distress; and interventions and support strategies. These themes provide a comprehensive overview of the ways in which moral distress has been described in the literature.

##### 3.1.2.1 Theme 1: Factors Contributing to Moral Distress

Studies identified a range of organisational, interpersonal, and demographic factors that contribute to the development and intensity of moral distress among ED nurses. Three subthemes were identified: protective factors, which lessened the intensity of moral distress; exacerbating factors, which heightened its impact; and demographic influences on moral distress.

###### 3.1.2.1.1 Subtheme 1.1: Protective Factors

Positive workplace conditions were consistently associated with lower moral distress. Greater job satisfaction, higher engagement, and supportive organisational climates appeared to buffer the impact of distress (Clark et al. 2021; Kızıltepe and Koç 2025). Some contextual features of the ED were also protective. For example, the episodic nature of patient contact, compared with the longer‐term relationships typical of ICU, was described as less emotionally burdensome (Robinson and Stinson 2016). Similarly, nurses working in both ambulance and ED settings valued the opportunity to provide focused, one‐on‐one, person‐centred care during transport, which reduced their sense of distress (Jiménez‐Herrera and Axelsson 2015).

###### 3.1.2.1.2 Subtheme 1.2: Exacerbating Factors

Organisational constraints were consistently identified as major contributors to moral distress. Nurses described inadequate staffing, unsafe working conditions, and restrictive institutional or legal requirements as key barriers that prevented them from providing ethically appropriate care (Babamohamadi et al. 2021; Clark et al. 2022; Jalali et al. 2019; Li et al. 2024; Zavotsky and Chan 2016). Within these contexts, a recurrent theme of interprofessional conflict emerged, particularly when nurses perceived physicians' orders for tests or treatments to be unnecessary, heightening distress (Jiménez‐Herrera and Axelsson 2015; Topan et al. 2023; Zavotsky and Chan 2016). Interpersonal contributors to moral distress were also evident, including relational tensions and concerns about compromised care when family members were absent (Robinson and Stinson 2016).

###### 3.1.2.1.3 Subtheme 1.3: Demographic Influences

The relationship between demographic characteristics and moral distress was inconsistent across studies. Older age, longer clinical experience, and resilience developed through practice were linked with lower distress and more effective coping (Babamohamadi et al. 2021; Clark et al. 2022; Kızıltepe and Koç 2025; Topan et al. 2023). However, two studies (Jalali et al. 2019; Zavotsky and Chan 2016) found no significant difference between level of experience or age and moral distress. Higher educational attainment and extended tenure in the ED were sometimes associated with greater moral distress, potentially reflecting heightened ethical awareness and prolonged exposure to distressing events (Babamohamadi et al. 2021; Kızıltepe and Koç 2025; Topan et al. 2023).

##### 3.1.2.2 Theme 2: Situational Triggers of Moral Distress in Emergency Nurses

The literature consistently identified specific clinical and ethical situations that triggered moral distress in emergency nurses. These scenarios were often characterised by conflict between nurses' professional values and the care they were ‘required’ to provide.

End‐of‐life and futile care situations were among the most frequently reported causes of moral distress (Babamohamadi et al. 2021; Jiménez‐Herrera and Axelsson 2015; Kızıltepe and Koç 2025; Robinson and Stinson 2016; Topan et al. 2023). Nurses described feeling morally distressed when initiating lifesaving interventions that were perceived as only prolonging the dying process, or when resuscitation was performed despite advanced directives being documented, or a lack of patient or family consent for care interventions (Babamohamadi et al. 2021; Robinson and Stinson 2016; Topan et al. 2023; Trautmann et al. 2015; Zavotsky and Chan 2016).

Resource related dilemmas were also identified as a common trigger of moral distress. High patient acuity and overcrowding were identified as a trigger, as they required emergency RNs to prioritise critically ill patients at the expense of lower‐acuity individuals, generating moral distress over the perceived inequality of care distribution (Clark et al. 2022). This form of moral distress was not associated with the act of triage prioritisation, but the ongoing resource constraints that limited nurses' capacity to provide care after triage decisions had been made. Notably, no studies mentioned triage as a point of moral distress. Inadequate equipment and cost‐driven restrictions on treatment were similarly described as situations that contributed to nurses' moral distress as nurses felt unable to deliver appropriate care (Clark et al. 2021; Jalali et al. 2019; Kızıltepe and Koç 2025).

Furthermore, the depersonalisation of care also emerged as a trigger for emergency nurses' moral distress. Participants from the study by Jiménez‐Herrera and Axelsson (2015) reported that emergency care practice sometimes reduced the patient to a diagnosis or a body part, fragmenting the caring process and, in turn, eroding opportunities for person‐centred holistic care.

##### 3.1.2.3 Theme 3: The Impact of Moral Distress on Emergency Nurses

Moral distress was consistently reported to have significant and multifaceted impacts on emergency nurses, shaping both their professional practice and their personal wellbeing. The consequences of moral distress appear to extend beyond the immediate clinical encounter, affecting nurses both professionally and personally. Impacts were reported at three levels: professional outcomes, personal wellbeing, and individual coping strategies.

###### 3.1.2.3.1 Subtheme 3.1: The Impact of Moral Distress on Emergency Nurses Professional Lives

Moral distress was consistently associated with negative consequences for emergency nurses' professional lives, particularly in relation to job satisfaction, engagement, and retention. Quantitative studies demonstrated significant associations between higher levels of moral distress and lower workplace engagement, reduced job satisfaction, and increased intent to leave (Clark et al. 2021; Topan et al. 2023). Similarly, moral distress was significantly higher in nurses who had considered leaving or had previously left a position due to moral distress (Trautmann et al. 2015; Zavotsky and Chan 2016). Qualitative findings reinforced these patterns, with participants describing how moral distress led them to disengage, consider leaving, or feel hardened over time in ways that ultimately impacted both themselves and the organisations they worked for (Robinson and Stinson 2016).

###### 3.1.2.3.2 Subtheme 3.2: The Impact of Moral Distress on Emergency Nurses Personal Lives

Moral distress was reported to have a profound impact on the personal lives and well‐being of emergency nurses, often extending beyond the workplace into their emotional and social relationships. Qualitative findings described this in terms of changes in persona (Robinson and Stinson 2016). To manage these experiences, nurses adopted a range of coping strategies. Adaptive strategies included positive reframing, acceptance, humour, planning, and seeking social support from colleagues, friends, and family (Kızıltepe and Koç 2025; Zavotsky and Chan 2016). Such approaches were perceived to mitigate, at least temporarily, the emotional burden of distress. However, maladaptive coping was also reported, including denial, behavioural disengagement, emotional suppression, inappropriate joking, hostility, and substance use (Robinson and Stinson 2016; Zavotsky and Chan 2016). These strategies were often framed as necessary defence mechanisms in the moment but were acknowledged by participants as contributing to longer‐term emotional harm and professional disengagement.

##### 3.1.2.4 Theme 4: Interventions and Support Strategies

While most studies focused on the causes and experiences of moral distress, several papers identified or suggested strategies that may help mitigate its impact on emergency nurses. These strategies clustered into three broad categories: organisational, educational, and ethical/psychological supports.

Organisational strategies were the most frequently discussed. Creating safe staffing levels, reducing workload pressures, and ensuring adequate resources were identified as key measures to reduce distress and enable nurses to deliver the standard of care they considered appropriate (Clark et al. 2021; Li et al. 2024). Retaining experienced nurses, valuing staff contributions, and fostering a supportive workplace culture were also emphasised as practical approaches for lowering moral distress and improving workplace engagement (Clark et al. 2022).

Educational strategies were highlighted as another important avenue for intervention. Several studies pointed to the role of ethics training, reflective workshops, and resilience‐building programs in equipping nurses with skills to navigate morally challenging situations (Clark et al. 2021; Topan et al. 2023; Ziba et al. 2022).

Ethical and psychological support strategies were also identified. Jiménez‐Herrera and Axelsson (2015) called for clearer ethical guidelines to support nurses in end‐of‐life decision‐making, particularly in resuscitation contexts. Other studies emphasised the importance of providing structured psychological support, stress management interventions, and environments where nurses feel empowered to raise ethical concerns without fear of reprisal (Li et al. 2024; Ziba et al. 2022).

## 4 Discussion

This review aimed to explore the extent, range, and nature of the published literature on moral distress among emergency nurses. Overall, the evidence base remains limited, with relatively few studies focusing specifically on ED nursing contexts compared with intensive care or other settings. Nevertheless, the included studies provide important insights into both the experience and impact of moral distress and the situational and systemic causes that underpin it.

The majority of the studies measuring moral distress to date have been conducted in non‐emergency contexts, such as ICU, oncology, or general hospital settings. Instruments such as the MDS and MDS‐R, may not capture the nuances of moral distress in the ED setting, whereas the broader MMD‐HP is more likely to capture this. Furthermore, differences between ED and general healthcare systems in the global community also presents a challenge for comparison. Differences in healthcare systems and country of origin further appears to shape moral distress risk with a systematic review and meta‐analysis undertaken by Alimoradi et al. (2023), identifying that nurses working in developing countries have higher rates of moral distress than those working in developed countries. This aligns with the findings of this scoping review, where studies from Iran (Babamohamadi et al. 2021; Jalali et al. 2019) identified moderate to high moral distress levels, and Topan et al. (2023) from emerging economy Turkey, identified that, although moral distress levels in their sample were low, paediatric emergency nurses reported relatively higher moral distress scores then nurses caring for paediatric patients in mixed emergency departments. Quantitative studies conducted within the United States healthcare system (Clark et al. 2022; Trautmann et al. 2015; Zavotsky and Chan 2016) consistently demonstrate the presence of moral distress among ED nurses and suggest it is closely associated with system level and patient‐related stressors.

Although the quantitative and mixed methods studies in the scoping review applied existing instruments to emergency department populations, ED‐specific psychometric validation of both inter‐rater reliability and validity was only identified in four studies: two MDS‐R (Babamohamadi et al. 2021; Li et al. 2024), one MMD‐HP (Clark et al. 2021), and one MDS (Kızıltepe and Koç 2025). Two studies, both of which use the MDS (Jalali et al. 2019; Ziba et al. 2022), cite a previous study undertaken by Omran et al. (2008) for testing the validity and reliability of the tool; however, the cited study was completed on general nurses in private hospitals, not within an ED context, and no measure of reliability is stated.

Studies varied in how moral distress was reported and interpreted, including reporting all three measurable aspects of moral distress (Babamohamadi et al. 2021; Topan et al. 2023; Zavotsky and Chan 2016), these being the frequency, intensity, and the composite scores; studies that only reported composite scores (Clark et al. 2021; Trautmann et al. 2015), and studies that did not report moral distress scores (Li et al. 2025). While higher scores indicate greater moral distress, there is yet to be consensus on a threshold for categorising levels of distress. Consequently, apparent differences in moral distress levels across settings or populations may reflect methodological variation and interpretation rather than substantive differences in moral distress.

Across the studies, moral distress was consistently described as a significant and multifaceted experience for emergency nurses. It was often triggered when nurses perceived a conflict between their professional values and the care they were required to deliver. These findings align with research from ICU and critical care nurses, that identified feelings of moral distress when providing care that participants perceived as futile (Gagnon and Kunyk 2022; Hiler et al. 2018; Jetten and Allard 2025), or when organisational constraints, such as inadequate staffing or working with staff that are not perceived to be as competent, limited their ability to act in accordance with ethical standards (Wolf 2019; Woods 2020).

In contrast to the ICU and other critical care environments where moral distress is often associated with prolonged end of life decision‐making, sustained exposure to futile care and ongoing relationships with patients and families (Qu et al. 2026), the ED context introduces additional factors that shape the nature of moral distress for ED nurses. Limited continuity of care, rapid patient turnover, the need for constant reprioritisation of care and frequent handovers mean ED nurses often identify unmet clinical, emotional, or social needs of their patients, and where they are unable to meet these needs or see the outcomes of their care (Walters et al. 2023). Although unmet clinical, emotional, and social needs of patients are reflected across commonly used moral distress instruments, these needs are not always measured explicitly or consistently. Instead, they are often embedded within items related to inadequate care, unsafe staffing, resource constraints, or unrelieved suffering. Furthermore, as the three main instruments that were used within the identified studies within this scoping review (the MDS, MDS‐R, and the MMD‐HP) were developed and tested in an American context, clinical, emotional, and social needs of patients may be interpreted differently across global contexts and healthcare systems, potentially influencing how moral distress is experienced and reported. Items such as ‘be unable to provide optimal care due to pressures from administrators or insurers’ from the MMD‐HP (Epstein et al. 2019) and or ‘provide better care for those that can afford to pay than those that cannot’ (Corley et al. 2001, p254) may not be directly relevant to nurses working in healthcare environments where emergency care is free at the point of treatment, for example, within the United Kingdom or Australian public hospitals settings.

Furthermore, diagnostic uncertainty is a defining feature of emergency nursing practice and represents a key contextual factor that distinguishes EDs from other acute care environments, including the ICU. Emergency RNs are frequently required to act in the absence of a confirmed diagnosis, often relying on incomplete information, evolving clinical presentation, and time‐critical decision‐making. This uncertainty may heighten moral distress as ED RNs must balance the risk of participating in care that causes unnecessary suffering or does not adequately relieve pain or symptoms, an item explicitly identified as a cause of moral distress on the MMD‐HP, but not in the original MDS. The MDS was originally written for measuring moral distress in ICU nurses, where patients are typically admitted following diagnosis, with more clearly defined care trajectories. However, moral distress research in the ICU context (Corley et al. 2001; Hamric et al. 2012; Qu et al. 2026; Witton et al. 2023) frequently highlights futile care as a top five cause of moral distress, which is similar to studies in this review, including Babamohamadi et al. (2021), Jiménez‐Herrera and Axelsson (2015) and Robinson and Stinson (2016) where participants identified providing futile care as a leading cause of their moral distress.

Emergency Department nurses work in contexts where demand frequently exceeds available staff, available beds, required equipment and time, creating ongoing challenges related to the allocation of limited resources (Clark et al. 2022; Jalali et al. 2019). Beyond acuity and unpredictability, the ethical climate of ED practice is often shaped by sustained resource scarcity and ongoing prioritisation of care decisions that require clinicians to balance competing patient needs within constrained systems. This climate is further influenced by perceptions of non‐urgent or inappropriate ED use, which clinicians report as negatively impacting their capacity to deliver timely and equitable care to patients with acute needs (Simsek and Gürsoy 2016). These conditions contribute to an ethical environment in which moral tension arises not from isolated dilemmas, but from the routine necessity to make trade‐offs between fairness, access and quality of care.

These findings resonate with data linking moral distress to perceived threats to care quality (DeKeyser Ganz and Berkovitz 2012). The professional impacts were described in the literature as considerable, with studies identifying associations between moral distress and reduced job satisfaction, lower workplace engagement, and higher turnover intentions (Trautmann et al. 2015; Zavotsky and Chan 2016). These impacts are consistent with evidence linking moral distress to subsequent burnout and intent to leave the profession (Jetten and Allard 2025). Rather than acting as a single antecedent, moral distress may function as a reinforcing process, interacting with workload stressors and organisational culture to accelerate burnout over time. The ED is particularly susceptible to this pattern due to resource scarcity in relation to staffing, limited continuity of care, and constant reprioritisation, resulting in ethical tensions that may not be resolved before the next demand arrives. The cumulative effect appears to create a feedback loop, where distress contributes to burnout and burnout further predisposes nurses to distress.

Demographic influences also shaped experiences of moral distress, though findings were inconsistent. The Transition Shock Theory conceptualised by Duchscher (2009) frames early practice for new graduate (inexperienced) nurses as a turbulent and stressful process. This is similar to the studies included in this review, which suggest that older age and longer tenure were associated with lower distress and more effective coping, possibly reflecting resilience gained through experience. However, literature on early career nurse experience is limited in this review, a finding supported by a recent integrative review by Loyd et al. (2023). Taken together, these findings suggest that targeted supports prior to graduation of nursing students and during the first 5 years of practice may be critical for reducing distress and preventing attrition.

These findings align with and extend the broader literature on nurse moral distress, suggesting that interventions must be multi‐level. Organisational measures such as safe staffing, supportive ethical climates, and strengthened nurse–physician relationships are required. At the same time, targeted supports for early‐career nurses, along with educational and resilience‐building strategies, may help mitigate vulnerability during the transition period.

## 5 Implications for Emergency Nursing Practice, Education and Leadership

Moral distress has been identified to be largely shaped by system‐level constraints, indicating that organisational response must go beyond individual resilience‐based approaches. Maintaining appropriate skill mix during periods of high demand, alongside transition support for newly graduated and early career nurses, should aim to reduce vulnerability through clear escalation pathways, supportive mentorship, and workload parameters. Integrating explicit discussion of moral distress, moral injury, and burnout may better prepare nurses for working within the ED and support earlier recognition and intervention.

Leadership practices remain central to shaping ethical climates. Leaders who promote open communication without reprisals and respond transparently to concerns may help validate nurses' moral agency and mitigate distress (Clark et al. 2022; Robinson and Stinson 2016; Zavotsky and Chan 2016). Future research should prioritise intervention studies in emergency nursing that integrate these organisational and individual approaches and include longitudinal designs to capture long‐term outcomes. Expanding the evidence base in diverse emergency contexts will also be important for developing globally relevant solutions.

## 6 Limitations

This scoping review has several limitations. The included studies were mapped but not appraised for methodological quality, which limits the ability to comment on the robustness of the evidence. Only studies published in English were included, which may have excluded relevant research published in other languages. By excluding review articles, the analysis may have overlooked broader insights and syntheses that could have provided additional contextual insight.

Beyond these procedural limitations, several substantive limitations were evident in the evidence base itself. The literature was dominated by research from Western, high‐income countries, with comparatively fewer studies from developing or diverse healthcare systems. This limits the global generalisability of findings, given the known differences in organisational structures, staffing models, resource availability, and sociocultural understandings of ethical practice across international settings.

Finally, considerable heterogeneity was evident in how moral distress was conceptualised and measured across studies. Multiple instruments were used to capture overlapping but not equivalent constructs, all of which were developed within the American healthcare context. This variability limits comparability across contexts and may contribute to differences in reported prevalence, causes, and outcomes of moral distress.

## 7 Conclusion

This scoping review mapped the extent, range and nature of published research examining moral distress among emergency nurses. The literature was predominantly characterised by cross‐sectional quantitative studies and single‐site qualitative investigations, largely conducted in high‐income Western healthcare systems. Across studies, moral distress was most commonly measured using the MDS, MDS‐R or MMD‐HP, with relatively little psychometric validation for ED contexts.

Across methodological approaches, moral distress was consistently described as a multifaceted phenomenon associated with emotional exhaustion, reduced job satisfaction, increased intent to leave, and perceived compromises to care quality. The review identified recurring sources of moral distress, including organisational and system‐level constraints, resource scarcity, perceived futility of care, and the depersonalisation of care, inherent in ED environments. Demographic, professional, and contextual factors shaped both the intensity and interpretation of moral distress, highlighting its situated and relational nature within emergency practice.

Despite an expanding evidence base, this review identified substantial and persistent knowledge gaps. Considerable heterogeneity in conceptual framing and measurement tools limited cross‐study comparability and synthesis, reinforcing concerns of conceptual ambiguity. Notably, the literature remains heavily focused on identifying causes of moral distress, with a notable absence of evaluated interventions, organisational strategies, or leadership approaches specifically tailored to emergency nursing contexts. Longitudinal research is particularly lacking, limiting understanding of how moral distress accumulates, resolves or transforms across career stages and how repeated exposure to ethically challenging situations may contribute to moral residue, moral injury or longer‐term professional harm.

These findings underscore the need for further conceptual and empirical work to refine definitions of moral distress and related constructs, strengthen measurement consistency and support the development of targeted, context‐responsive interventions. Addressing these gaps is essential to advancing theoretical clarity, improving research comparability, and informing meaningful organisational and policy responses to moral distress in emergency nursing.

## Author Contributions

C.W. conceived the study topic and developed the overall review concept. C.W. designed the protocol methodology in accordance with JBI guidance. C.W. drafted the initial search strategy with input from a university librarian. M.M. and C.Y. refined the final search strategy. C.W. and M.M. independently screened all records and full texts in Covidence (Veritas Health Innovation 2025). C.Y. adjudicated any conflicts. C.W. and M.M. conducted data extraction, charting, and analysis, with discrepancies resolved through discussion or, if needed, arbitration by C.Y. C.W. prepared the first draft of the manuscript. All authors critically reviewed, revised, and approved the final manuscript.

## Funding

The authors have nothing to report.

## Conflicts of Interest

The authors declare no conflicts of interest.

## Supporting information


**Data S1:** Preferred Reporting Items for Systematic reviews and Meta‐Analyses extension for Scoping Reviews (PRISMA‐ScR) Checklist.

## Data Availability

Data sharing not applicable to this article as no datasets were generated or analysed during the current study.
